# Poor nutrition doubles post-COVID-19 syndrome risk in cancer patients: insights from a Chinese multicentre study

**DOI:** 10.3389/fnut.2024.1479918

**Published:** 2024-10-30

**Authors:** Liangyuan Zhang, Haihang Yu, Jianzhou Yang, Rila Su, Jiaqi Zhang, Rongbiao Zeng, Yajie Liu, Lei Zhang, Junjie Xu

**Affiliations:** ^1^Clinical Research Academy, Peking University Shenzhen Hospital, Shenzhen, Guangdong, China; ^2^Department of Epidemiology, China Medical University, Shenyang, Liaoning, China; ^3^Department of Public Health and Preventive Medicine, Changzhi Medical College, Changzhi, Shanxi, China; ^4^Cancer Center of Inner Mongolia People’s Hospital, Hohhot, Inner Mongolia, China; ^5^Johns Hopkins Bloomberg School of Public Health, Baltimore, MD, United States; ^6^Department of Radiation Therapy, Peking University Shenzhen Hospital, Shenzhen, Guangdong, China; ^7^Department of Oncology, Peking University Shenzhen Hospital, Shenzhen, Guangdong, China

**Keywords:** cancer patients, COVID-19, nutritional status, post-COVID-19 syndrome, multicentre cross-sectional study

## Abstract

**Background:**

Since 2019, approximately 760 million SARS-CoV-2 cases have been reported globally, with post-COVID-19 syndrome posing significant challenges for cancer patients due to their immunosuppressed status and poor nutritional conditions. The role of nutritional status in influencing their infection risk and post-COVID-19 outcomes remains unclear, underscoring the need for targeted research and strategies.

**Objective:**

To investigate the impact of baseline nutritional status on SARS-CoV-2 infection and the risk of post-COVID-19 syndrome in cancer patients.

**Methods:**

A multicenter cross-sectional study was conducted from December 2022 to June 2023 in four tertiary hospitals across China. Cancer inpatients aged 18 years and older were enrolled and classified into two groups based on their Nutritional Risk Screening (NRS) scores. The correlation between SARS-CoV-2 infection, post-COVID-19 syndrome and nutritional status were analyzed using multivariable logistic regression.

**Results:**

Among 834 eligible cancer patients, 10.8% were in the high nutritional risk group (NRS ≥ 3). The prevalence of SARS-CoV-2 infection was 58.8% (95% confidence interval, CI: 56.8–60.8%), and post-COVID-19 syndrome was 21.0% (95% CI: 10.4–14.4%). After adjusting for confounding factors, the high nutritional risk group had a significantly higher prevalence of post-COVID-19 syndrome compared to the low nutritional risk group (32.7% vs. 19.5%, AOR: 2.37, 95% CI: 1.23–4.54, *p* = 0.010). However, no significant difference in SARS-CoV-2 infection rates was found between the two groups (61.1% vs. 58.5%, AOR: 1.12, 95% CI: 0.70–1.80; *p* = 0.634).

**Interpretation:**

Poor baseline nutritional status in cancer patients is associated with a higher prevalence of post-COVID-19 syndrome, providing preliminary information on post-COVID-19 syndrome in this population. These findings underscore the importance of adequate nutritional management in cancer patients, particularly during pandemic recurrences.

## 1 Introduction

Since 2019, approximately 760 million cases of SARS-CoV-2 infection have been reported globally, resulting in 6.9 million deaths (1). Post-COVID-19 syndrome, characterized by persistent fatigue, cognitive sequelae, and respiratory impairment, is prevalent among SARS-CoV-2 survivors and presents substantial challenges for specific patient populations, including cancer patients (2). Cancer patients are often immunosuppressed due to their disease and treatments such as radiotherapy and chemotherapy, which exacerbate their nutritional deterioration (3–5).

A study have shown that during the pandemic, the prevalence of malnutrition among cancer patients in the Brazil hospital reached approximately 34.6% (6), nearly eight times greater than that in the general population of Brazil (4.7%) (7). A UK study revealed that individuals with weaker immunity and poorer nutritional status have a significantly increased risk of SARS-CoV-2 infection and post-COVID-19 syndrome (8). Moreover, cancer patients suffering from vitamin D deficiency face even higher risks and mortality rates (9). These findings suggest that poor nutritional status is a key factor in the clinical outcome of SARS-CoV-2 infection. The Prognostic Nutritional Index (PNI) is commonly used to assess the nutritional status of cancer patients because it has been demonstrated to be particularly predictive of outcomes in oncological settings (10), making it highly relevant for assessing the risk and prognosis of malnutrition-related complications in patients infected with SARS-CoV-2. However, systematic studies on the relationships between Nutritional Risk Screening 2002 (NRS-2002) scores, nutritional status, and SARS-CoV-2 infection rates in cancer patients are still lacking. Existing research and clinical data suggest that nutritional status may be closely linked to the risk of SARS-CoV-2 complications and recovery duration in cancer patients, although the specific impacts remain unclear (11, 12). The importance of nutritional status in cancer patients’ clinical outcomes has been well-documented in various contexts (13, 14). Malnutrition has been associated with increased treatment toxicity, reduced response to therapy, and poorer quality of life in cancer patients (13–16). In the context of infectious diseases, including COVID-19, nutritional status plays a crucial role in maintaining immune function and promoting recovery. However, the specific relationship between nutritional status and the risk of SARS-CoV-2 infection and post-COVID-19 syndrome in cancer patients remains understudied. The recurring waves of the COVID-19 pandemic have highlighted the need for a better understanding of risk factors for severe disease and long-term complications in cancer patients.

While age, comorbidities, and cancer status have been identified as significant risk factors (17, 18), the role of nutritional status in determining outcomes in cancer patients infected with SARS-CoV-2 requires further investigation. This knowledge gap is particularly important given the high prevalence of malnutrition in cancer patients and the potential for targeted nutritional interventions to improve outcomes. To address this research gap, we conducted a multicenter cross-sectional study across four provinces in China. Our aim was to investigate the influence of the nutritional status of cancer patients on the risk of SARS-CoV-2 infection and post-COVID-19 syndrome. This study seeks to provide scientific evidence for targeted COVID-19 prevention and control strategies for cancer patients in potential future recurrences or pandemics of variant strains. By elucidating these relationships, we hope to inform clinical practice and guide the development of nutritional interventions to improve outcomes for cancer patients during the ongoing COVID-19 pandemic and beyond.

## 2 Methods

### 2.1 Study design and participants

**Figure 1:**
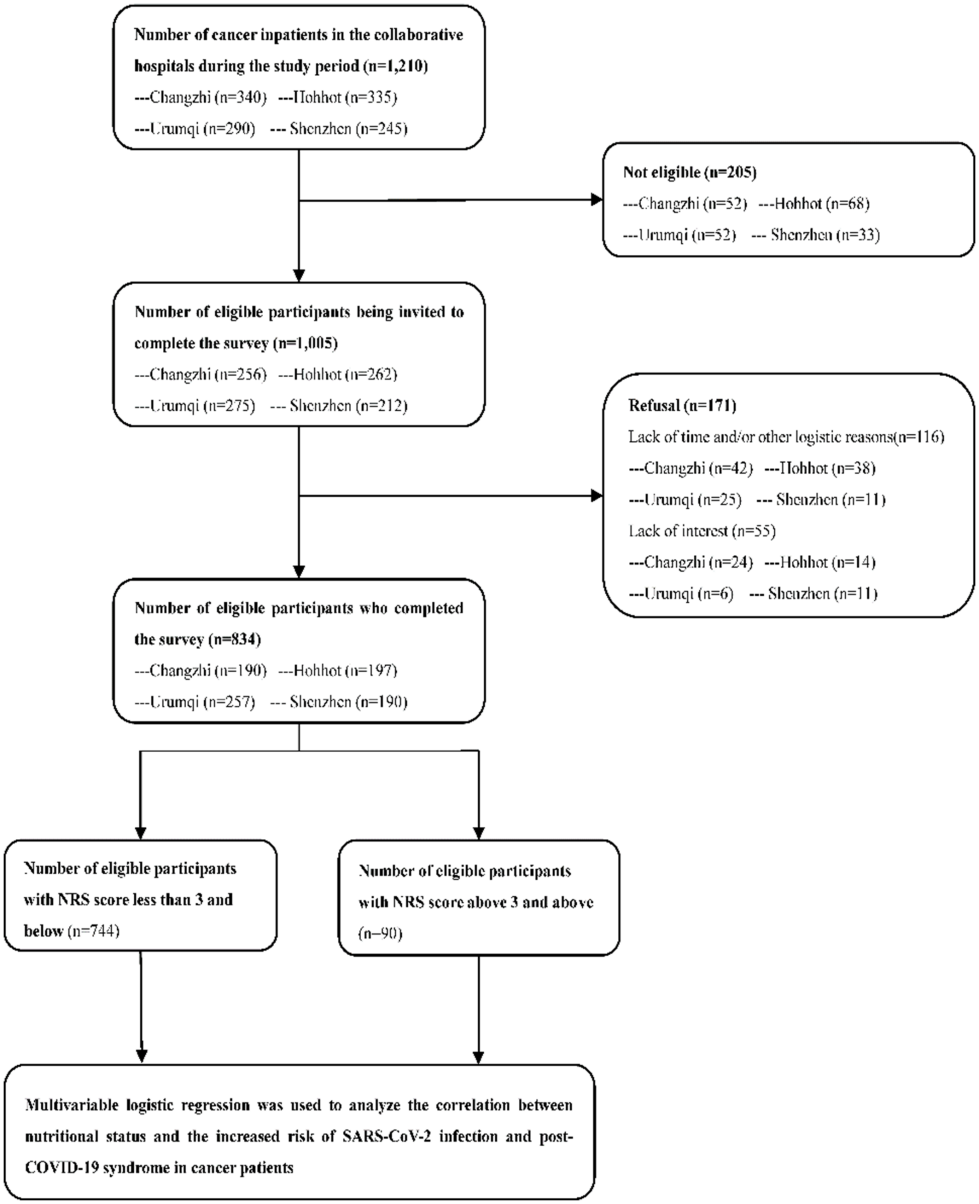
The data collection procedures of this study.

This multicentre cross-sectional study was conducted from December 2022 to June 2023 in four tertiary hospitals across four provinces (Guangdong, Shanxi, Xinjiang and Inner Mongolia) in China. The hospitals included Shanxi Heping Hospital (affiliated with Changzhi Medical College), Inner Mongolia People’s Hospital in North China, the First Affiliated Hospital of Xinjiang Medical University in Northwest China, and Guangdong Peking University Shenzhen Hospital in South China. Participants were recruited from these hospitals. The study employed a convenience sampling method by selecting the largest hospital in each region and used a consecutive enrollment approach during the study period to recruit cancer patients from these hospitals until the required sample size was achieved (Figure 1).

The eligibility criteria for participants were as follows: (1) aged 18 years and above, (2) cancer inpatients at the four participating hospitals during the study period, and (3) expressed willingness to participate by signing the informed consent form. The exclusion criteria included individuals who were diagnosed with lymphoma, leukemia, or mental illness; who were undergoing treatment for mental illness; and who had communication difficulties with the researchers. Hematological malignancy patients were excluded from this study due to China’s previous vaccination guidelines, which restricted them from receiving the COVID-19 vaccine, resulting in most of them remaining unvaccinated.

### 2.2 Data collection

This study examined the impact of different nutritional statuses on the prevalence of SARS-CoV-2 infection and post-COVID-19 syndrome among cancer patients in China after the comprehensive lifting of pandemic restrictions on December 5, 2022. The data were collected by medical staff responsible for patient recruitment and screening in the oncology departments of the participating hospitals. Patients who met the specific inclusion and exclusion criteria mentioned above were selected. After providing informed consent, patient medical records at admission and self-report questionnaires completed on the “Jinshuju” platform were collected. The information gathered included sociodemographic data, cancer type, comorbidities, and treatment modalities. All procedures strictly followed the principles of the Declaration of Helsinki and were approved by the Ethics Review Committee of Changzhi Medical College (protocol code RT2022027). Access to medical records and questionnaires was granted after receiving written informed consent from participants.

### 2.3 Disease diagnosis and relevant criteria

Within 48 h of admission, nutritional risk was assessed using the Nutritional Risk Screening 2002 (NRS2002) tool recommended by the European Society for Clinical Nutrition and Metabolism (ESPEN) and the Chinese Society for Parenteral and Enteral Nutrition (CSPEN) for evaluating nutritional status in hospitalized cancer patients. This tool was specifically chosen due to its validated sensitivity and specificity in identifying malnutrition risks among cancer patients, offering a robust framework for early intervention (19, 20). Disease severity (scored 1 to 3 for cancer patients based on stress metabolism caused by the disease), impaired nutritional status [scored 0 to 3 based on weight loss, body mass index (BMI), and food intake], and age (1 point added if ≥70 years old) were assessed. The total score ranges from 0 to 7, with a score ≥3 indicating nutritional risk and a score <3 indicating no nutritional risk. The assessment was performed by two specially trained experts, and patients self-reported their weight (unit: kilogram) and height (unit: centimeter) (19, 20).

The diagnosis of SARS-CoV-2 infection was based on the clinical reported history of positive result for SARS-CoV-2 detected by reverse transcription-polymerase chain reaction (RT–PCR) on a nasopharyngeal swab or the patient’s self-reported related symptoms (21, 22). The diagnosis of post-COVID-19 syndrome was based on the ICD-10-CM diagnostic code (U09.9 post-COVID-19 condition, unspecified) (23). Patients were diagnosed with post-COVID-19 syndrome if they continued to experience fatigue, shortness of breath, dizziness, loss of taste or smell, cough, brain fog, or psychological symptoms 1 month after SARS-CoV-2 infection. This diagnosis was made after considering the patient’s COVID-19 history, reviewing self-reported symptoms and hospital medical records, and excluding other possible causes (2). Experienced oncologists with more than 10 years of clinical experience were involved in the comprehensive assessment to distinguish symptoms related to cancer and its treatment from those related to long-term COVID-19.

### 2.4 Sample size calculation

Based on the literature review and a small-scale preliminary study, the SARS-CoV-2 infection rate was estimated to be 20% in the non-malnourished group and 35% in the malnourished group (24). To detect this difference, with *α* = 0.05 (two-sided) and 1-*β* = 0.8, the Fleiss (25) design formula was used for the calculation, resulting in a requirement of at least 136 patients per group. Additionally, considering potential response rate, the sample size was increased by 20%, ultimately requiring approximately 170 patients per group. Therefore, the overall sample size needed was 340 patients.

### 2.5 Data analysis

Multiple imputation was used to ensure the completeness and accuracy of the analysis results for missing data (≤30%), variables with more than 30% missing data were excluded from the analysis. Continuous variables with a normal distribution, such as age, are described as the mean ± standard deviation, while categorical variables, such as gender, ethnicity, and education level, are described as frequencies and percentages. The primary outcome measures, including the SARS-CoV-2 infection rate and the prevalence of post-COVID-19 syndrome, were compared using chi-square tests, and odds ratios (ORs) and their 95% confidence intervals (CIs) were calculated to assess the associations between malnutrition and infection and post-COVID-19 syndrome. For the multivariable logistic regression, variables that remained significant in the preliminary analysis were further adjusted. The selection of potential confounders such as age, gender, cancer type, and treatment modality were based on their established associations with the outcomes in prior research. We employed a backward stepwise selection process to refine the model, which systematically identifies and retains only those variables that contribute significantly to the model, thus enhancing the model’s fit and reducing potential confounder effects. This method ensures that the final model includes only variables that have a statistically significant impact on the outcome, minimizing the risk of spurious associations due to confounding. Adjusted odds ratios (AORs) and their 95% confidence intervals (CIs) were calculated to elucidate the independent effects of each variable. All the statistical analyses were performed using SPSS (version 26.0, IBM Corp.) and R (version 4.0.2) software, with the significance level set at *p* = 0.05, and all the tests were two-sided.

## 3 Results

### 3.1 Baseline characteristics

During the study period, 1,210 cancer inpatients were recruited from the four study sites, of which 1,005 (83.1%) met the inclusion criteria. After providing informed consent, 834 participants completed the NRS-2002 and questionnaires survey. Based on the NRS scores, 90 participants (10.8%) were categorized as having high nutritional risk (NRS score ≥3), and 744 (89.2%) were categorized as having low nutritional risk (NRS score <3).

Compared to the NRS score <3 group, the NRS score ≥3 group had a significantly greater mean age (65.3 ± 10.7 years vs. 57.3 ± 12.8 years, *p* < 0.001), a greater proportion of males (57.8% vs. 45.7%, *p* = 0.03), and a greater proportion of individuals with a BMI <18.5 (26.7% vs. 10.2%, *p* < 0.001) (Table 1).

**Table 1:** Characteristics of participants according to different NRS score groups.

Sociodemographic characteristics	Total	Nutritional risk screening		*p-*value
		<3 points	≥3 points	
	*N* = 834	*N* = 744	*N* = 90	
Age (y)	58.12 ± 12.8	57.3 ± 12.8	65.3 ± 10.7	**<0.001**
Male sex	392 (47.0%)	340 (45.7%)	52 (57.8%)	**0.03**
BMI (kg/m^2^)				**<0.001**
<18.5	100 (12.0%)	76 (10.2%)	24 (26.7%)	
18.5–23.9	461 (55.3%)	410 (55.1%)	51 (56.7%)	
24–27.9	220 (26.4%)	207 (27.8%)	13 (14.4%)	
28+	53 (6.4%)	51 (6.9%)	2 (2.2%)	
Ethnicity				0.687
Han majority	740 (88.7%)	659 (88.6%)	81 (90.0%)	
Other ethnic minorities	94 (11.3%)	85 (11.4%)	9 (10.0%)	
Education level				0.373
Junior high or below	528 (63.3%)	466 (62.6%)	62 (68.9%)	
Senior high or equivalent	173 (20.7%)	155 (20.8%)	18 (20.0%)	
College and above	133 (15.9%)	123 (16.5%)	10 (11.1%)	
Relationship status				0.299
Single/divorced/widowed	85 (10.2%)	73 (9.8%)	12 (13.3%)	
Married	749 (89.8%)	671 (90.2%)	78 (86.7%)	
Employment status				0.069
Full-time	141 (16.9%)	132 (17.7%)	9 (10.0%)	
Part-time/self-employed/unemployed/retired/students	693 (83.1%)	612 (82.3%)	81 (90.0%)	
Behavioral lifestyle characteristics				
Smoking				0.525
No smoking	467 (56.0%)	416 (55.9%)	51 (56.7%)	
Non-smoker, exposed to secondhand smoke	137 (16.4%)	126 (16.9%)	11 (12.2%)	
Past	155 (18.6%)	138 (18.5%)	17 (18.9%)	
Present	75 (9.0%)	64 (8.6%)	11 (12.2%)	
Frequency of alcohol consumption (times per week)				0.786
Never consumes alcohol	627 (75.2%)	561 (75.4%)	66 (73.3%)	
<7	183 (21.9%)	161 (21.6%)	22 (24.4%)	
≥7	24 (2.9%)	22 (3.0%)	2 (2.2%)	
Duration of sleep (h)				**0.005**
<7	333 (39.9%)	285 (38.3%)	48 (53.3%)	
7–9	468 (56.1%)	432 (58.1%)	36 (40.0%)	
>9	33 (4.0%)	27 (3.6%)	6 (6.7%)	
Cancer related characteristics				
Type of cancer				
Lung cancer	167 (20%)	145 (19.5%)	22 (24.4%)	0.269
Gastric cancer	74 (8.9%)	59 (7.9%)	15 (16.7%)	**0.007**
Breast cancer	127 (15.2%)	122 (16.4%)	5 (5.6%)	**0.011**
Colorectal cancer	106 (12.7%)	93 (12.5%)	13 (14.4%)	0.601
^a^Other cancers	376 (45.1%)	338 (45.4%)	38 (42.2%)	0.564
Cancer status				**0.005**
Cured	156 (18.7%)	150 (20.2%)	6 (6.7%)	
Not cured	346 (41.5%)	309 (41.5%)	37 (41.1%)	
^b^Others	332 (39.8%)	285 (38.3%)	47 (52.2%)	
Types of cancer treatment				
Chemotherapy only	382 (45.8%)	340 (45.7%)	42 (46.7%)	0.862
Radiotherapy only	179 (21.5%)	171 (23.0%)	8 (8.9%)	**0.003**
Immunotherapy only	145 (17.4%)	133 (17.9%)	12 (13.3%)	0.285
^c^Others	354 (42.4%)	302 (40.6%)	52 (57.8%)	**0.002**
Presence of chronic disease conditions				**0.013**
No	465 (55.8%)	426 (57.3%)	39 (43.3%)	
Yes	369 (44.2%)	318 (42.7%)	51 (56.7%)	
Vaccination status				0.096
Unvaccinated	192 (23.0%)	165 (22.2%)	27 (30.0%)	
Vaccinated	642 (77.0%)	579 (77.8%)	63 (70.0%)	
One dose	34 (4.1%)	26 (3.5%)	8 (8.9%)	
Two doses	152 (18.2%)	135 (18.1%)	17 (18.9%)	
Three doses	440 (52.8%)	403 (54.2%)	37 (41.1%)	
Four or more doses	16 (1.9%)	15 (2.0%)	1 (1.1%)	

NRS, nutritional risk screening 2002; BMI, body mass index; OR, odds ratio; ^a^Other cancers include esophageal, bladder, kidney, liver, ovarian, nasopharyngeal cancer etc.; ^b^Others include cancer not detected, undiagnosed and recurrence after remission; ^c^Other treatments include chemotherapy plus radiotherapy, immunotherapy plus chemotherapy or radiotherapy, endocrine and traditional Chinese medicine (TCM), targeted therapy and completed treatment. Except for the variables ‘Presence of Chronic Disease Conditions’ and ‘Sociodemographic Characteristics’, the definitions of the remaining variables pertain to the period before the comprehensive lifting of pandemic restrictions in China on December 5, 2022. Bold values represent *p*-values less than 0.05, indicating statistical significance.

The NRS score ≥3 group also had a greater proportion of patients with a sleep duration <7 h (53.3% vs. 38.3%, *p* = 0.005), a greater proportion with unhealed cancer (52.2% vs. 38.3%, *p* = 0.005), and a greater proportion receiving combined cancer treatments (57.8% vs. 40.6%, *p* = 0.002). They also had more proportion of chronic diseases (56.7% vs. 42.7%, *p* = 0.013). Although the group with NRS scores ≥3 had similar rate of COVID-19 vaccination (70.0% vs. 77.8%, *p* = 0.096). There were no significant differences in sociodemographic characteristics such as ethnicity, education level, marital status, or employment status (all *p* > 0.05) (Table 1).

### 3.2 Relationships between baseline characteristics, SARS-CoV-2 infection status, and post-COVID-19 syndrome events

A total of 58.8% (95% CI: 56.8–60.8%) of cancer patients experienced SARS-CoV-2 infection before the study. Univariate logistic regression analysis revealed that compared with individuals of Han ethnicity, individuals of other ethnic minorities (OR: 0.30, 95% CI: 0.20–0.48) and who received radiation therapy only (OR: 0.70, 95% CI: 0.50–0.98) were less likely to have SARS-CoV-2 infection. In contrast, patients who only received chemotherapy were more likely to have SARS-CoV-2 infection (OR: 1.64, 95% CI: 1.24–2.17). At the same time, cancer patients with a college degree or above had a greater risk of SARS-CoV-2 infection than did those with lower education levels (OR: 1.85, 95% CI: 1.23–2.78), and those who had never been vaccinated against COVID-19 were also more likely to have SARS-CoV-2 infection than were those who had received at least one dose of the vaccine (OR: 1.18, 95% CI: 1.02–1.38) (Figure 1).

A total of 12.4% (95% CI: 10.4–14.4%) of cancer patients experienced the post-COVID-19 syndrome. Compared to females, males (OR: 1.78, 95% CI: 1.13–2.80) were more likely to develop post-COVID-19 syndrome. Additionally, compared to those with lower education levels, cancer patients with a college degree or above (OR: 2.44, 95% CI: 1.42–4.19) and those exposed to secondhand smoke (OR: 2.13, 95% CI: 1.24–3.68) were more likely to develop post-COVID-19 syndrome. Breast cancer patients were also more likely to develop post-COVID-19 syndrome than were patients with other cancer types (OR: 2.24, 95% CI: 1.32–3.79), and patients who received only radiotherapy (OR: 0.45, 95% CI: 0.23–0.87) were more likely to develop post-COVID-19 syndrome (Table 2).

**Table 2:** Association of baseline characteristics with COVID-19 infection status and post-COVID-19 syndrome status.

Characteristics	COVID-19 infection status			Post-COVID-19 syndrome status		
	Prevalence % (*n*/*N*)	OR (95%CI)	*P-*value	Prevalence % (*n*/*N*)	OR (95%CI)	*P-*value
Sociodemographic characteristics						
Proportion of male sex	56.1% (220/392)	1.23 (0.93, 1.62)	0.146	15.9% (35/220)	1.78 (1.13, 2.80)	**0.013**
BMI (kg/m^2^)			0.493			0.658
<18.5	60.0% (60/100)	Ref.		25.0% (15/60)	Ref.	
18.5–23.9	57.0% (263/461)	0.89 (0.57, 1.38)		19.8% (52/263)	0.74 (0.38, 1.43)	
24–27.9	62.7% (138/220)	1.12 (0.69, 1.82)		20.3% (28/138)	0.76 (0.37, 1.56)	
28+	54.7% (29/53)	0.81 (0.41, 1.58)		27.6% (8/29)	1.14 (0.42, 3.11)	
Ethnicity			**<0.001**			0.492
Han majority	62.0% (459/740)	Ref.		21.4% (98/459)	Ref.	
Other ethnic minorities	33.0% (31/94)	0.30 (0.20, 0.48)		16.1% (5/31)	0.71 (0.27, 1.89)	
Education level			**0.012**			**0.002**
Junior high or below	55.7% (294/528)	Ref.		15.6% (46/294)	Ref.	
Senior high or equivalent	59.5% (103/173)	1.17 (0.83, 1.67)		27.2% (28/103)	2.01 (1.18, 3.44)	
College and above	69.9% (93/133)	1.85 (1.23, 2.78)		31.2% (29/93)	2.44 (1.42, 4.19)	
Relationship status			0.494			0.964
Single/divorced/widowed	55.35% (47/85)	Ref.		21.3% (10/47)	Ref.	
Married	59.1% (443/749)	1.17 (0.75, 1.84)		21.0% (93/443)	0.98 (0.47, 2.05)	
Employment status			0.435			0.620
Full-time	61.7% (87/141)	Ref.		23.0% (20/87)	Ref.	
Part-time/self employed/unemployed/retired/students	58.2% (403/693)	0.86 (0.60, 1.25)		20.6% (83/403)	0.87 (0.50, 1.51)	
Behavioral lifestyle characteristics						
Smoking			0.931			**0.020**
No smoking	58.9% (275/467)	Ref.		19.3% (53/275)	Ref.	
Non-smoker, exposed to secondhand smoke	60.6% (83/137)	1.07 (0.73, 1.58)		33.7% (28/83)	2.13 (1.24, 3.68)	
Past	56.8% (88/155)	0.92 (0.64, 1.32)		15.9% (14/88)	0.79 (0.42, 1.51)	
Present	58.7% (44/75)	0.99 (0.60, 1.63)		18.2% (8/44)	0.93 (0.41, 2.12)	
Frequency of alcohol consumption (times per week)			0.646			0.325
Never consumes alcohol	59.6% (374/627)	Ref.		19.8% (74/374)	Ref.	
<7	56.3% (103/183)	0.87 (0.62, 1.22)		26.2% (27/103)	1.44 (0.87, 2.39)	
≥7	54.2% (13/24)	0.80 (0.35, 1.81)		15.4% (2/13)	0.74 (0.16, 3.40)	
Duration of sleep (h)			0.692			0.597
<7	57.1% (190/333)	Ref.		23.2% (44/190)	Ref.	
7–9	60.0% (281/468)	1.13 (0.85, 1.50)		19.9% (56/281)	0.83 (0.53, 1.29)	
>9	57.6% (19/33)	1.02 (0.50, 2.11)		15.8% (3/19)	0.62 (0.17, 2.23)	
Cancer related characteristics						
Type of cancer						
Lung cancer	58.1% (97/167)	0.97 (0.69, 1.36)	0.844	16.5% (16/97)	0.70 (0.39, 1.25)	0.224
Gastric cancer	60.8% (45/74)	1.10 (0.67, 1.79)	0.706	15.6% (7/45)	0.67 (0.29, 1.55)	0.348
Breast cancer	63.0% (80/127)	1.23 (0.84, 1.82)	0.292	33.8% (27/80)	2.24 (1.32, 3.79)	**0.003**
Colorectal cancer	61.3% (65/106)	1.13 (0.74, 1.72)	0.566	23.1% (15/65)	1.15 (0.62, 2.14)	0.662
^a^Other cancers	56.4% (212/376)	0.84 (0.64, 1.10)	0.208	18.4% (39/212)	0.75 (0.48, 1.18)	0.214
Cancer status			**0.018**			0.665
Cured	55.8% (87/156)	Ref.		23.0% (20/87)	Ref.	
Not cured	64.5% (223/346)	1.44 (0.98, 2.11)		22.0% (49/223)	0.94 (0.52, 1.70)	
^b^Others	54.2% (180/332)	0.94 (0.64, 1.38)		18.9% (34/180)	0.78 (0.42, 1.46)	
Current treatment for cancer						
Chemotherapy only	65.2% (249/382)	1.64 (1.24, 2.17)	**0.001**	23.7% (59/249)	1.39 (0.90, 2.16)	0.141
Radiotherapy only	52.0% (93/179)	0.70 (0.50, 0.98)	**0.038**	11.8% (11/93)	0.45 (0.23, 0.87)	**0.016**
Immunotherapy only	58.6% (85/145)	0.99 (0.69, 1.43)	0.972	20.0% (17/85)	0.93 (0.52, 166)	0.800
^c^Others	55.6% (197/354)	0.80 (0.61, 1.06)	0.118	22.8% (45/197)	1.20 (0.77, 1.86)	0.417
Presence of chronic disease conditions			0.308			0.275
No	57.2% (266/465)	Ref.		19.2% (51/226)	Ref.	
Yes	60.7% (224/369)	1.16 (0.88, 1.53)		23.2% (52/224)	1.28 (0.83, 1.97)	
Vaccination status			**0.021**			0.152
Unvaccinated	51.6% (99/192)	Ref.		26.3% (26/99)	Ref.	
Vaccinated	60.9% (391/642)	0.85 (0.73, 0.98)		19.7% (77/391)	0.75 (0.51, 1.10)	
One dose	58.8% (20/34)	0.99 (0.75, 1.33)		35.0% (7/20)	0.58 (0.31, 1.09)	
Two dose	65.1% (99/152)	0.88 (0.77, 1.01)		19.2% (19/99)	1.12 (0.72, 1.75)	
Three dose	59.8% (263/440)	0.96 (0.86, 1.08)		10.6% (51/263)	1.18 (0.84, 1.67)	
Four or more doses	56.3% (9/16)	1.05 (0.68, 1.62)		0.0% (0/9)	NA	

BMI, body mass index; OR, odds ratio; ^a^Other cancers include esophageal, bladder, kidney, liver, ovarian, nasopharyngeal cancer etc.; ^b^Others include cancer not detected, undiagnosed and recurrence after remission; ^c^Other treatments include chemotherapy plus radiotherapy, immunotherapy plus chemotherapy or radiotherapy, endocrine and traditional Chinese medicine (TCM), targeted therapy and completed treatment. Except for the variables ‘Presence of Chronic Disease Conditions’ and ‘Sociodemographic Characteristics’, the definitions of the remaining variables pertain to the period before the comprehensive lifting of pandemic restrictions in China on December 5, 2022. Bold values represent *p*-values less than 0.05, indicating statistical significance.

### 3.3 The correlation between nutritional risk score and SARS-CoV-2 infection

**Figure 2:**
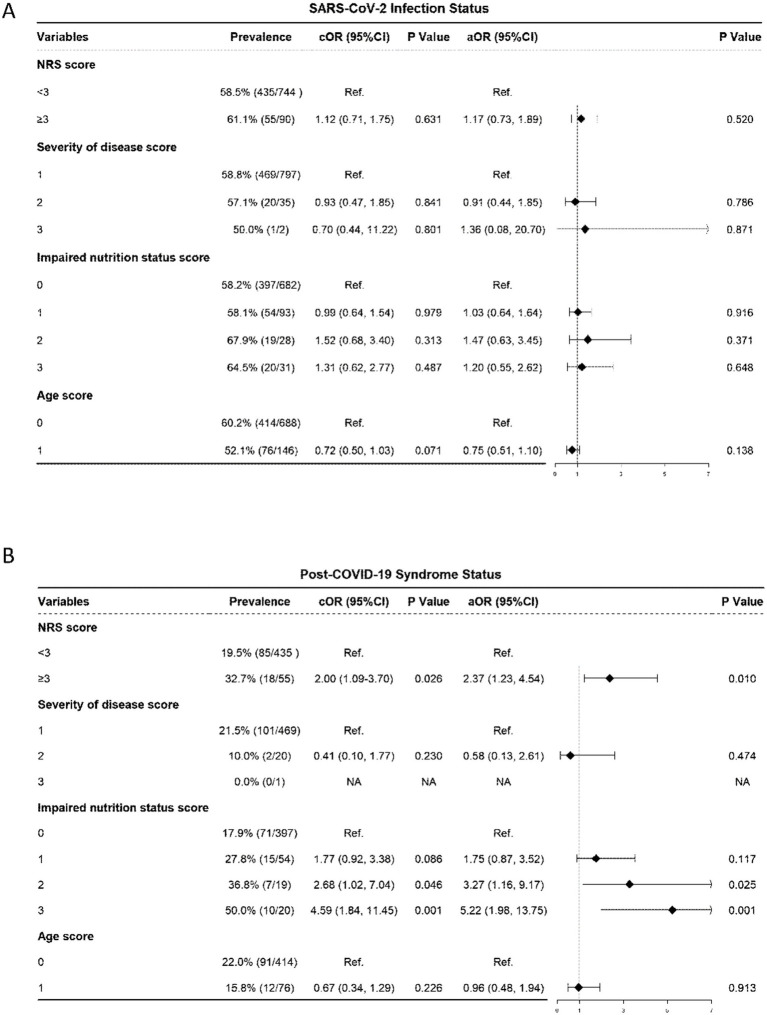
Association of NRS with SARS-CoV-2 infection Status and Post-COVID-19 Syndrome Status. **(A)** SARS-CoV-2 infection Status. **(B)** Post-COVID-19 Syndrome Status. NRS, nutritional risk screening 2002, the NRS score includes the score of severity of disease, impaired nutrition status and age of the patient; OR, odds ratio; aOR, adjusted odds ratio, odds ratios adjusted for significant sociodemographic characteristics listed in Table 2 and incorporated together into the same model; CI: confidence interval.

Patients with an NRS score <3, the prevalence of SARS-CoV-2 was 58.5%; for patients with an NRS score ≥3, the prevalence was slightly greater, at 61.1%. After baseline adjustment, multivariable logistic regression analysis showed that NRS score and prevalence of SARS-CoV-2 infection revealed that for the risk of SARS-CoV-2 infection did not significantly increase for patients with NRS score ≥3 (AOR: 1.17, 95% CI: 0.73–1.89; *p* = 0.520) than the patients with NRS score ≤3 group (Figure 2).

In terms of disease severity score, there was no statistically significant difference in the prevalence of SARS-CoV-2 infection between patients with a score of 1 and those with a score of 2 (58.8% vs. 57.1%) or between patients with a score of 2 and 3 (*p* > 0.05); regarding impaired nutritional status score, the prevalence of SARS-CoV-2 infection showed no statistically significant difference between patients with a score of 0 and 1 (58.2% vs. 58.1%) or between patients with a score of 2 and 3 (67.9% vs. 64.5%) (all *p* > 0.05); for age score, there was no statistically significant difference in the prevalence of SARS-CoV-2 infection between patients with a score of 0 and 1 (60.2% vs. 52.1%, *p* = 0.138) (Figure 2A).

### 3.4 The correlation between nutritional risk score and post-COVID-19 syndrome

**Figure 3:**
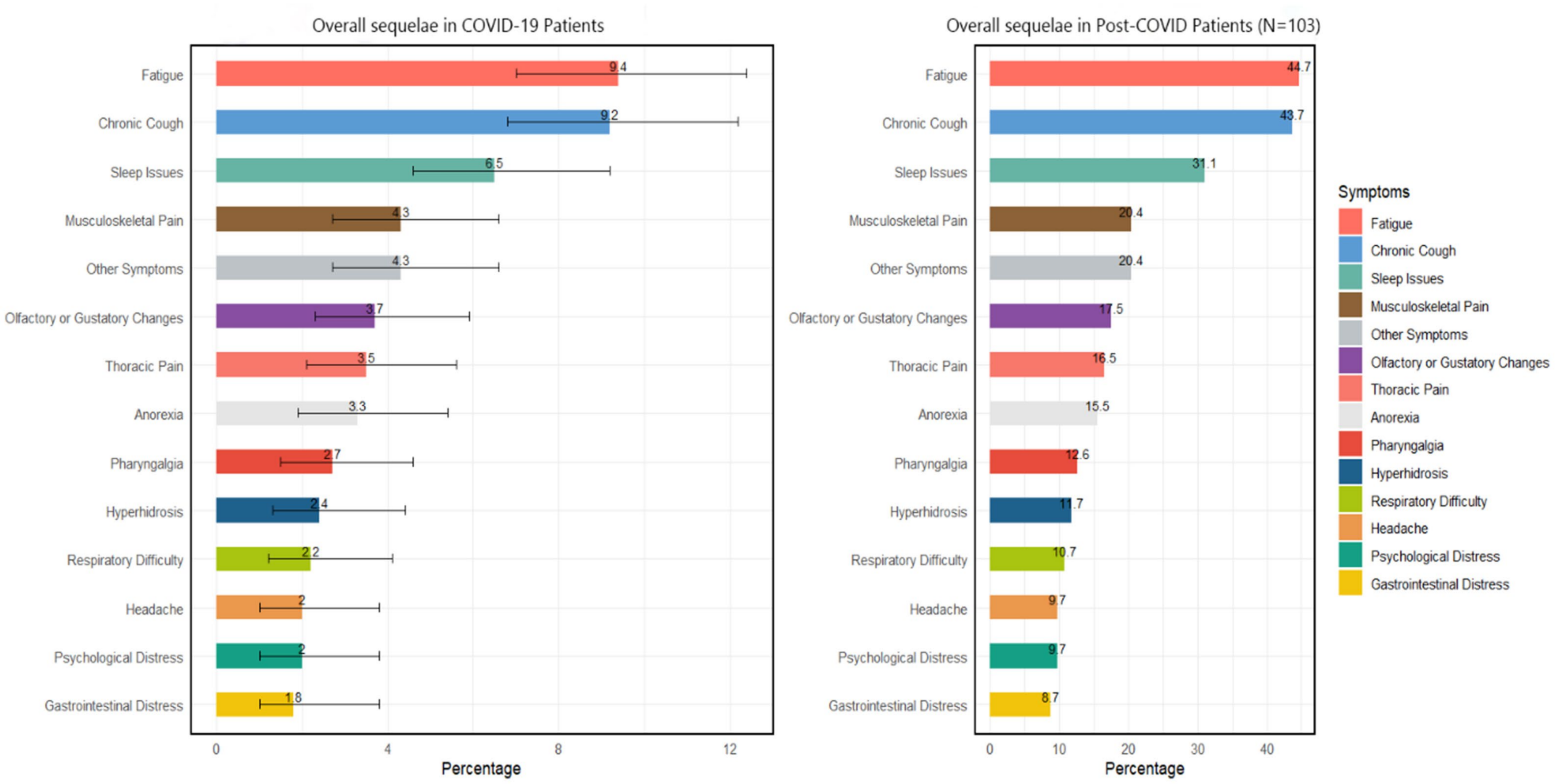
Overall COVID-19 sequelae according to COVID-19 patients and post-COVID patients. Other symptoms include skin issues, brain fog, persistent fever, alopecia, nausea, etc.

Among 490 self-reported SARS-CoV-2-infected cancer patients, 21.0% (95% CI: 19.0–23.0%) experienced post-COVID-19 syndrome symptoms. An evaluation of the symptom composition of 103 post-COVID-19 syndrome patients revealed that the most common symptoms were fatigue and chronic cough (44.7 and 43.7%, respectively). Moreover, 31.1% of the participants had sleep problems, and 20.4% had musculoskeletal pain. Smell or taste changes, chest pain, and loss of appetite had similar proportions, all approximately 15% (Figure 3).

According to the multivariable logistic regression analysis of nutritional risk score (NRS) and post-COVID-19 syndrome status, patients with an NRS score <3 had a 19.5% of post-COVID-19 syndrome, while those with an NRS score ≥3 had a 32.7% post-COVID-19 syndrome. After adjusting for baseline confounding factors, the risk of developing post-COVID-19 syndrome for patients with an NRS score ≥3 significantly increased than the counterpart group (AOR: 2.37, 95% CI: 1.23–4.54; *p* = 0.010) (Figure 2B).

In terms of disease severity and age, there were no significant differences (all *p* > 0.05) in the prevalence of post-COVID-19 syndrome (Figure 2B). However, regarding impaired nutritional status score, the prevalence rate of post-COVID-19 syndrome for patients with a score of 0 was 17.9%; as the nutritional status score increased, the prevalence rate of post-COVID-19 syndrome showed an upwards trend: 27.8% for patients with a score of 1 (AOR: 1.75, 95% CI: 0.87–3.52, *p* = 0.117); the prevalence rate significantly increased to 36.8% for patients with a score of 2 (AOR: 3.27, 95% CI: 1.16–9.17, *p* = 0.025); and among patients with a score of 3, the proportion of post-COVID-19 syndrome was as significantly high as 50.0% (AOR: 5.22, 95% CI: 1.98–13.75, *p* = 0.001) (Figure 2B).

## 4 Discussion

Our study provides important insights into the relationships between nutritional status, and the clinical outcomes of SARS-CoV-2 infection, and post-COVID-19 syndrome in cancer patients. The findings suggest that poor baseline nutritional status in cancer patients is associated with a higher prevalence of post-COVID-19 syndrome but not with an increased risk of SARS-CoV-2 infection. These results have significant implications for the management of cancer patients during the ongoing COVID-19 pandemic and potential future reoccurrence of outbreaks.

We found no significant difference in SARS-CoV-2 infection rates between patients with high and low nutritional risk scores, suggesting that nutritional status may not be a primary determinant of infection risk in this population. This finding contrasts with some previous studies that have suggested a link between poor nutritional status and increased susceptibility to infectious diseases. The lack of association in our study could be due to several factors. First, the lack of a significant association between nutritional status and SARS-CoV-2 infection in this study may be due to asymptomatic cases in some cancer patients and the absence of routine SARS-CoV-2 testing, which could have impacted the analysis. Second, the high overall infection rate in our study may have masked any subtle differences between nutritional risk groups. Third, other risk factors, such as cancer type, treatment modality, and exposure to healthcare settings, may have played a more dominant role in determining infection risk. Additionally, considering that our sample primarily originated from hospital environments, this setting could also influence the observed high rates of infection. Future research needs to encompass a broader sample and consider the interactions between these variables to more accurately assess the relationship between nutritional status and SARS-CoV-2 infection risk.

In contrast to the infection risk, our study revealed a strong association between poor nutritional status and the development of post-COVID-19 syndrome in cancer patients. Cancer patients with high nutritional risk (NRS ≥3) were more than twice as likely to develop post-COVID-19 syndrome compared to those with low nutritional risk. This finding aligns with previous studies that have linked malnutrition to prolonged recovery and increased complications in various diseases (6, 24, 26). The association between nutritional status and post-COVID-19 syndrome may be explained by several mechanisms. Malnutrition can impair immune function, leading to a dysregulated immune response and prolonged inflammation (27, 28). Additionally, poor nutritional status may compromise tissue repair and regeneration processes, potentially exacerbating and prolonging the effects of SARS-CoV-2 infection (29, 30). The most common symptoms of post-COVID-19 syndrome in our study population were fatigue and chronic cough, followed by sleep problems and musculoskeletal pain. These symptoms are consistent with those reported in the general population, but may have a more significant impact on cancer patients due to their already compromised health status. The high prevalence of these symptoms underscores the need for comprehensive follow-up care for cancer patients who infected with acute SARS-CoV-2. Our findings also have important clinical implications. They underscore the importance of nutritional assessment and management in cancer care, particularly in the context of the ongoing COVID-19 pandemic. Early identification of patients at high nutritional risk could help guide targeted interventions to potentially reduce the risk of post-COVID-19 syndrome, improve overall outcomes and reduce mortality rate (31, 32). Such interventions might include nutritional supplementation, dietary counseling, and strategies to address specific nutritional deficiencies. The study also highlights the complex relationship between various demographic and clinical factors and COVID-19 outcomes in cancer patients. For instance, we found that patients with higher education levels were more likely to be infected with SARS-CoV-2, which may reflect differences in social behaviors or occupational exposures. Males and breast cancer patients were more likely to develop post-COVID-19 syndrome, suggesting that both gender and cancer type may influence long-term outcomes. These findings emphasize the need for personalized risk assessment and management strategies for cancer patients during the pandemic.

### 4.1 Strengths and limitations

Our study has several strengths, including its multicenter design, large sample size, and comprehensive assessment of nutritional status using the validated NRS-2002 tool. The inclusion of patients from different regions of China enhances the generalizability of our findings within the Chinese context. However, some limitations should be noted. The cross-sectional nature of the study precludes causal inferences, and longitudinal studies are needed to confirm the temporal relationship between nutritional status and post-COVID-19 syndrome. The reliance on self-reported COVID-19 history may have led to some misclassification, although we attempted to mitigate this by reviewing medical records and involving experienced clinicians in the assessment. Additionally, while our study focused on nutritional status as assessed by the NRS-2002, future research should consider more comprehensive measures of nutritional status, including biochemical markers and body composition analysis. This could provide a more nuanced understanding of the specific nutritional factors that influence COVID-19 outcomes in cancer patients. Furthermore, the study was conducted in China, and the findings may not be fully generalizable to other populations or healthcare systems. Replication of these findings in diverse populations and healthcare settings is warranted.

## 5 Conclusion

In conclusion, our study highlights the critical role of nutritional status in the development of post-COVID-19 syndrome among cancer patients. Poor nutritional status is strongly associated with the increased risk of developing long-term complications. These findings emphasize the need for routine nutritional screening and targeted interventions in cancer care, especially in the context of the ongoing pandemic. Healthcare providers should be aware of the increased risk of post-COVID-19 syndrome in cancer patients with poor nutritional status and implement appropriate monitoring and support strategies. Future research should focus on several key areas. First, prospective studies are needed to confirm the causal relationship between nutritional status and post-COVID-19 syndrome in cancer patients. Second, intervention studies should evaluate the efficacy of nutritional support strategies in reducing the risk and severity of post-COVID-19 syndrome. Lastly, research into the underlying mechanisms linking nutritional status to long-term COVID-19 outcomes could inform the development of targeted therapies.

## Data Availability

The raw data supporting the conclusions of this article will be made available by the authors, without undue reservation.
